# Intermittent treatment with farnesyltransferase inhibitor and sulforaphane improves cellular homeostasis in Hutchinson-Gilford progeria fibroblasts

**DOI:** 10.18632/oncotarget.19363

**Published:** 2017-07-18

**Authors:** Diana Gabriel, Dinah Dorith Shafry, Leslie B. Gordon, Karima Djabali

**Affiliations:** ^1^ Department of Dermatology, Epigenetics of Aging, TUM School of Medicine, Technische Universität München, Garching-Munich, Germany; ^2^ Department of Pediatrics, Alpert Medical School of Brown University and Hasbro Children’s Hospital, Providence, RI, USA; ^3^ Boston Children’s Hospital and Harvard Medical School, Boston, MA, USA

**Keywords:** progerin, lamin, Ionafarnib, sulforaphane, autophagy, Gerotarget

## Abstract

Hutchinson-Gilford progeria syndrome (HGPS) is a rare genetic condition associated with mutations in the *LMNA* gene. This disease recapitulates some aspects of normal aging, such as hair loss, thin skin, joint stiffness, and atherosclerosis. The latter leads to heart attack or stroke that causes death at an average age of 14.6 years in children with HGPS. The typical *LMNA* mutation results in the production of a truncated prelamin A protein, progerin, that remains permanently farnesylated and abnormally associated with the nuclear envelope. Farnesyltransferase inhibitors (FTIs) reverse nuclear structure abnormalities that are characteristic of HGPS cells. The first clinical trial using the FTI, Ionafarnib, demonstrated some improvements in HGPS children and, in particular, showed a decrease in arterial stiffness. Recently, we reported that sulforaphane, an antioxidant derived from cruciferous vegetables, efficiently stimulates autophagy and enhances progerin clearance in HGPS fibroblasts. In the present study, we investigated the effect of combined lonafarnib and sulforaphane treartment in HGPS fibroblast cultures. We report that co-administration of both drugs exerts a synergistic and additive positive effect on autophagy activity but was cytotoxic to HGPS cells. In contrast, intermittent treatment with lonafarnib followed by sulforaphane separately and in repeated cycles rescued the HGPS cellular phenotype. We propose that intermittent treatment with FTI and SFN separately might be a promising therapeutic avenue for children with HGPS.

## INTRODUCTION

Hutchinson-Gilford progeria syndrome is a premature aging disorder linked to mutations in the lamin A gene [1, 2]. In most cases, the genetic basis is a cytosine to thymine substitution in exon 11 at position 1824 of *LMNA*. This point mutation leads to a novel cryptic splice site within exon 11, causing a 50-amino-acid deletion at the carboxy-terminal end of the prelamin A protein [3]. The normal prelamin A protein undergoes a series of posttranslational modifications to become mature lamin A protein: 1) The cysteine of the *CaaX* motif is farnesylated by farnesyltransferase; 2) The last three amino acids of the *CaaX* motif are cleaved off by RCE1 or ZMPSTE24; 3) The carboxyl-terminal farnesylcysteine is methylated by isoprenylcysteine carboxyltransferase (ICMT); and 4) The last 15 amino acids at the carboxyl-terminus of the protein including the farnesylcysteine are cleaved off by ZMPSTE24 [4, 5]. Due to the point mutation G608G, the cleavage site for ZMPSTE24 is missing, leading to the production of a permanently farnesylated protein that remains tightly anchored to the nuclear envelope [6]. This truncated prelamin A protein is called progerin and induces severe changes in nuclear morphology, chromatin organization, mitosis, and DNA replication [7-9]. Progerin toxicity is attributed, in part, to its farnesyl moiety. Therapeutics such as farnesyltransferase inhibitors, statins, and bisphosphonates that target the maturation steps of prelamin A in particular the prenylation have been intensively investigated [10]. Farnesyltransferase inhibitors (FTIs) have been shown to reverse the nuclear abnormalities caused by progerin [11-13]. HGPS cells treated with FTIs exhibited successful delocalization of progerin from the nuclear envelope to the nucleoplasm [5]. With this outcome, the efficacy of FTIs treatment in progeria mouse models was tested. Three FTI studies using progeria mouse models have shown substantial improvements in body weight, life span, and adipose tissue [5]. However, under these treatment conditions, nonfarnesylated prelamin A and nonfarnesylated progerin still accumulate and remain in the nucleus. Studies of nonfarnesylated progerin knock-in mice showed that progerin remained toxic in its nonfarnesylated form [14]. Additionally, the accumulation of nonfarnesylated normal prelamin A was shown to induce cardiomyopathy in mice [15]. The disruption of the lamin B1 and lamin B2 processing was observed after FTI treatment in fibroblast cells [16] and was linked to deficiencies in the DNA damage response [17]. These studies also indicated that FTI treatment did not reduce the levels of DNA damage nor ameliorate the DNA damage signaling response [18]. Nevertheless, the positive effects of FTI in mouse models of HGPS strongly supported the use of Ionafarnib in the first clinical trial, which showed improvements in body weight, bone rigidity and mineral density as well as a reduction in arterial stiffness in children with HGPS [5, 19].

Recently, we showed that isothiocyanate sulforaphane (SFN) ameliorates the HGPS cellular phenotype *in vitro* [20].

SFN is a plant-derived compound and is a well-known activator of the Nrf2 signaling pathway, which promotes the cell’s intrinsic defense system via the regulation of cytoprotective genes [21-23]. Hence, SFN possesses antioxidant activity, anti-cancer activity, and anti-genotoxicity as a chemopreventive agent [21, 22, 24]. The ability of SFN to enhance the degradation pathways led to increased progerin clearance in HGPS fibroblasts, and via its antioxidant activity, SFN induced decreases in reactive oxygen species and DNA damage levels [20, 25]. In this present study, we tested the effects of combining both FTI and SFN treatment in HGPS cells. We postulated that FTI treatment, by delocalizing progerin from the nuclear envelope, would facilitate its degradation via SFN-enhanced protein degradation systems. Using this strategy in HGPS fibroblasts, we show that intermittent treatments with FTI and SFN ameliorated the HGPS cellular phenotype and increased progerin clearance.

## RESULTS

### Treatment of HGPS fibroblasts with a combination of farnesyltransferase inhibitor (lonafarnib) and sulforaphane

We tested the hypothesis that FTI would delocalize progerin from the nuclear envelope by blocking progerin farnesylation and that the SFN-induced activation of autophagy would increase progerin protein degradation.

**Figure 1:**
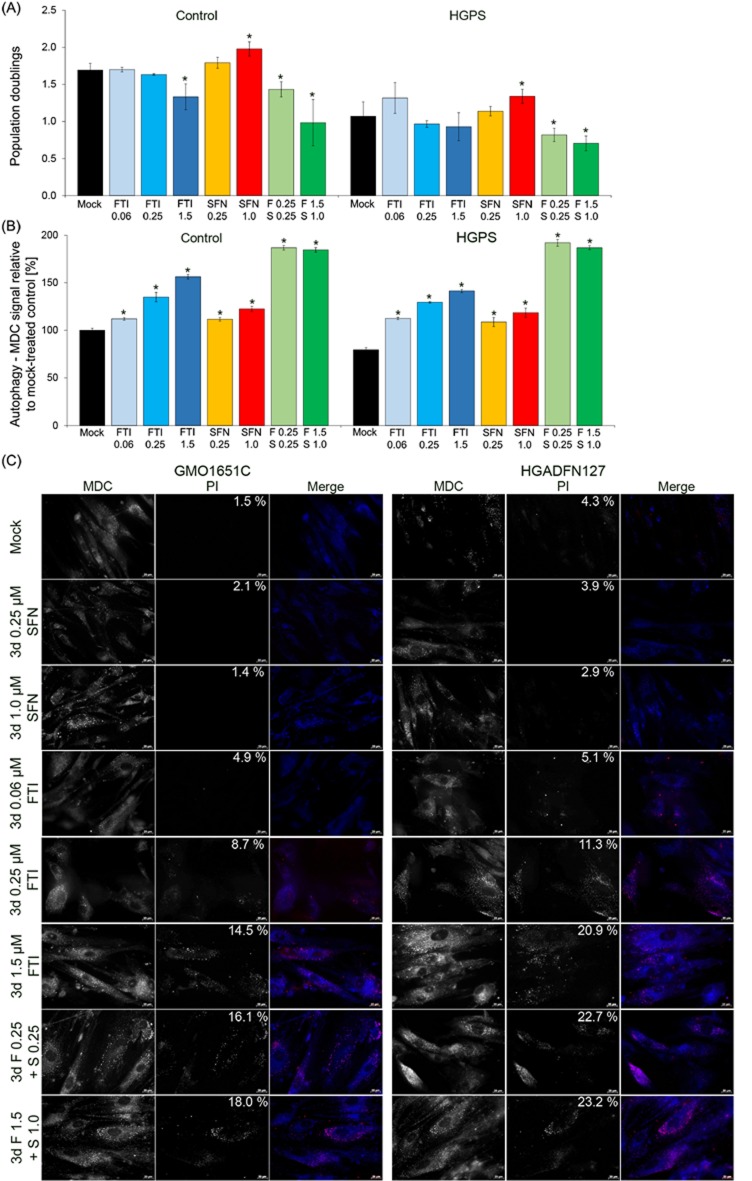
Effect of combined FTI and SFN on HGPS cells **A.** Population doublings were calculated as stated in Materials and Methods for control and HGPS fibroblasts. Cells were either treated with vehicle (DMSO), SFN (0.25 μM or 1.0 μM), FTI (0.5 μM, 1.0 μM, or 1.5 μM), or combined drugs at the following concentrations: 0.25 μM FTI plus 0.25 μM SFN or 1.5 μM FTI plus 1.0 μM SFN for a period of 3 days. **B.** The same cells as in (A) were used to measure the levels of autophagy vacuoles by monodansylcadaverine (MDC) as described in Materials and Methods. Data are expressed as the mean ± S.D. (*p-value ≤ 0.05; *n* = 3). **C.** Immunohistochemistry of control (GMO1651C) and HGPS live cells (HGADFN127) using antibodies against monodansylcadaverine (MDC, autophagic vacuoles, blue) and propidium iodide (PI, viability, red). The percentages of dead cells (PI positive cells) are indicated and were determined by direct counts of an average of 900 cells for each treatment. Cells were treated as stated in (A). Representative images are shown (*n* = 3). Scale bar: 20 μM.

In an earlier study, we showed that the combined daily treatment of FTI (lonafarnib, 1.5 μM) and SFN (1 μM) in HGPS cells induced high levels of cell death within 72 hours [20]. To further determine the extent to which these two compounds can be combined to restore HGPS cell homeostasis, we first investigated whether lowering the concentrations of both drugs could alleviate the cellular toxicity. We found that decreasing the FTI concentration to 0.06 μM slightly increased the growth rate of HGPS cells (*p* = 0.689; Figure 1A). Treatment of HGPS cells with SFN alone at 0.25 μM also promoted cell growth (*p* = 0.127; Figure 1A). When the cells were treated with both FTI and SFN at concentrations of 0.25 μM for a period of 3 days, the growth rates were significantly reduced in both control (*p* = 0.029) and HGPS cells (*p* = 0.030), which indicates that this combination was toxic (Figure 1A). Lowering the FTI concentration to 0.06 mM in combination with 0.25 μM SFN similarly decreased cell growth in the control (*p* = 0.00033) and HGPS (*p* = 0.0020) cells (Supplementary Figure 1A).

Previously, we showed that SFN activates autophagy [20]; therefore, we determined the levels of autophagy in both control and HGPS cells treated with FTI, SFN or a cocktail of both (Figure 1B). Autophagy activity was determined by measuring the levels of monodansylcadaverine (MDC), which labels autophagosomes as described in the Materials and Methods section. Treatment with FTI at 0.06 μM increased autophagy levels in control (*p* = 0.021) and HGPS (*p* = 0.009) cells after a treatment period of 3 days (Figure 1B). Autophagy levels were significantly increased progressively with the increased concentration of FTI or SFN treatment alone (Supplementary Figure 1B and Figure 1B). When cells were treated with a cocktail of FTI and SFN, the autophagy levels were much higher than that observed when the drugs were administered individually (Figure 1B). This finding indicates that treatment with both compounds had a synergistic and additive effect on autophagy activity in both control and HGPS cells (Figure 1B).

To further investigate why the cells treated with both compounds exhibited reduced growth, we performed immunohistochemistry to detect autophagosomes with monodansylcadaverine (MDC) and cell death with propidium iodide (PI) on living cells (Figure 1C). In the mock-treated control and HGPS cells, the signal for MDC was visible in the control cells and was weaker in the HGPS cells (Figure 1C, panel Mock). The percentages of PI-positive cells (signifying dead cells) are indicated on the PI images. The percentage of dead cells among the mock-treated HGPS cells (4.3%) was higher than that in the mock-treated control cells (1.5%). Increased levels of autophagosomes and cell death were observed in control and HGPS cells that were treated with FTI and SFN together at concentrations of 0.25 μM each (Figure 1C). At these concentrations the percentages of dead cells increased significantly in both control (16.1%) and HGPS (22.7%) cultures. Hence, PI-positive cells were brightly stained with MDC as shown in the merged images (Figure 1C, FTI 1.5 μM and combined drug treatments). This finding indicates that treatment with both drugs combined activated autophagy to levels that become toxic as indicated by the increased levels of cell death (Figure 1C).

To further explore the status of autophagy, we labeled the adaptor protein sequestosome 1 (SQSTM1/p62), a protein that recruits ubiquitinated proteins to autophagosomes and the lysosomal-associated membrane protein 2 (LAMP2) [26, 27] by immunofluorescence labeling (Supplementary Figure 2). Images showed an increased formation of p62- and LAMP2-positive punctuates in the cytoplasm of cells treated with FTI or SFN (Supplementary Figure 2A). Colocalization of both markers was more prominent in cells treated with both drugs (Supplementary Figure 2A). Western blot analysis confirmed that autophagy activity was enhanced in cells treated with both drugs as indicated by elevated levels of LC3-II and Lamp2 as well as the reduced levels of p62 protein, which together signified that the autophagic flux remained functional in FTI+SFN treated cells (Supplementary Figure 2B) [26]. Collectively, these findings show that combined treatment with FTI and SFN strongly activates autophagy and concomitantly reduces cell growth as well as increases cell death in normal and HGPS cultures. Thus, this drug cocktail is toxic to the cells.

### Defining the lowest concentrations of FTI and sulforaphane that still activate autophagy

**Figure 2:**
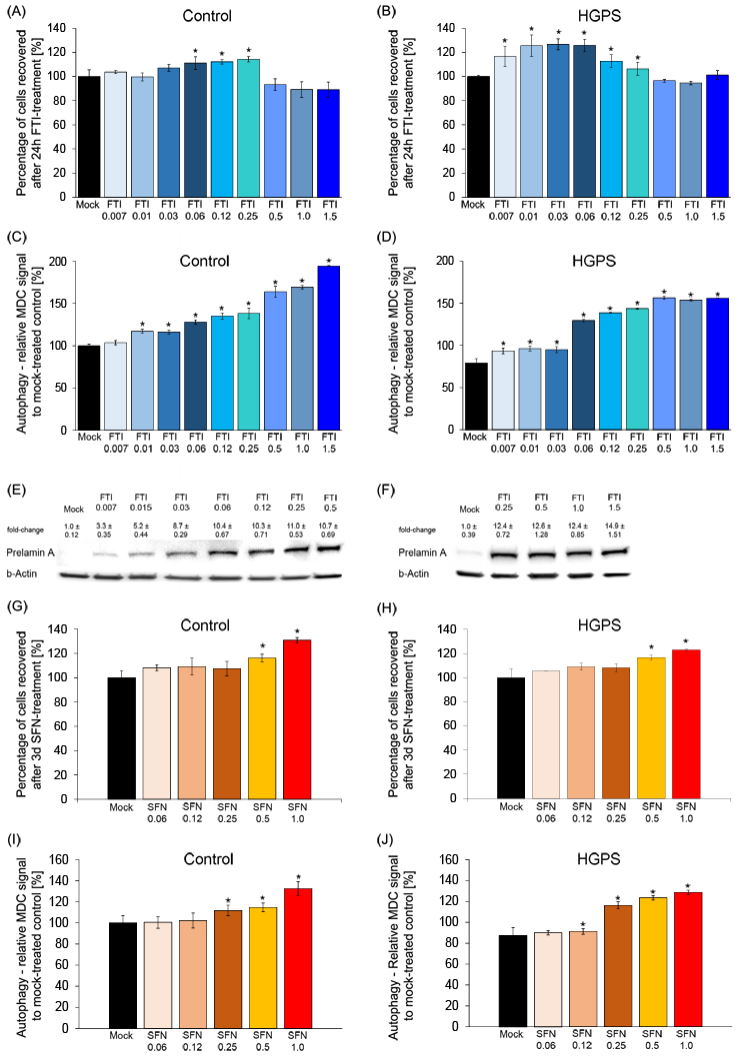
Defining the minimal FTI and SFN doses effective in HGPS cells **A.** Control cells were mock-treated (vehicle, DMSO) or treated for 24 hours with different concentrations of FTI, as indicated. The percentage of cells recovered after drug treatment was calculated relative to mock-treated cells. Data are expressed as the mean ± S.D. (*p-value ≤ 0.05; *n* = 5). **B.** HGPS cells were treated and analyzed as stated in (A). **C.** and **D.** Autophagy activity was measured as described in the Methods section for control and HGPS cells, as indicated. Cells were treated with different concentrations of FTI for 24 hours as indicated. The percentage of activity was calculated relative to mock-treated cells. Data are expressed as the mean ± S.D. (*p-value ≤ 0.05; *n* = 5). **E.** and **F.** Western blot of HGPS cells mock-treated or treated with different concentrations of FTI for 24 hours. Blot was probed with an anti-prelamin A antibody and b-Actin. Numbers above each band indicate the fold-change ± S.D. of prelamin A accumulation relative to mock-treated control. **G.** and **H.** Control and HGPS cells were mock-treated or treated with different concentrations of SFN daily for 3 days as indicated. The percentage of cells recovered after drug treatment was calculated relative to mock-treated cells and presented as the mean± S.D. (*p-value ≤ 0.05; *n* = 5). **I.** and **J.** The same cells and culture conditions as in (G and H) were used to measure the levels of autophagy in control (G) and HGPS (H) cells. Autophagy activity was determined using monodansylcadaverine (MDC). Data are expressed as the mean ± S.D. relative to mock-treated cells (*p-value ≤ 0.05; *n* = 5).

We tested decreasing concentrations of both compounds individually in control and HGPS cells (Figure 2). We determined that FTI at 0.06 μM maintained the viability of both cell types (Figure 2A, 2B) and activated autophagy in HGPS to a similar degree as in normal cells (Figure 2C, 2D). Cells treated with FTI accumulate nonfarnesylated prelamin A at a level that is proportional to the efficiency of farnesyltransferase inhibition, and therefore, in an FTI-dependent manner [4]. We determined the levels of prelamin A accumulation in HGPS cells treated with FTI at different concentrations for a period of 24 hours (Figure 2E, 2F). Prelamin A was detected and accumulated to a similar extent in HGPS cells treated with 0.06 µM to 0.5 µM of FTI and at an even higher level in cells treated with 1 µM FTI (Figure 2E, 2F). These studies indicated that treatment with FTI at a concentration of 0.06 µM was sufficient to block a fraction of prelamin A maturation as well as stimulate cell growth and autophagy activity in HGPS and normal cells. As 0.06 µM of FTI provided the best balance between cellular toxicity, autophagy and prelamin A accumulation, this concentration was selected for use in the subsequent studies.

Next, a similar titration of SFN on cell viability and autophagy activity was conducted (Figure 2G to 2J). As previously reported, daily treatment for a period of 3 days with 1μM SFN improved cell growth and activated autophagy to a similar range in both cell types [20]. Collectively, these results indicate that treatment with 0.06 μM FTI or 1μM SFN individually was sufficient to stimulate autophagy in normal and HGPS cells.

### Intermittent treatment with FTI and sulforaphane individually enhances progerin clearance in HGPS cells

**Figure 3:**
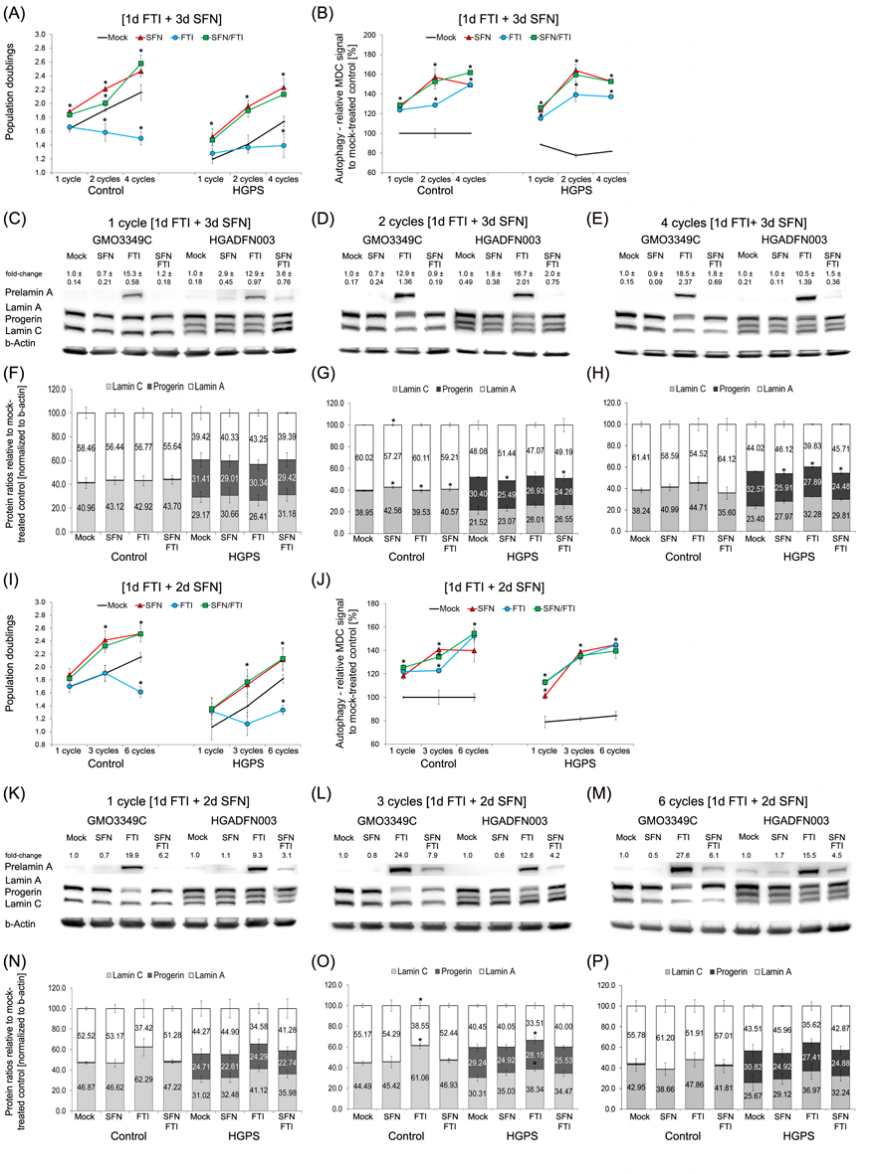
Intermittent treatment cycles with 1 day of FTI followed by 3 days of SFN enhance progerin clearance **A.** Population doublings were calculated as stated in Materials and Methods for control and HGPS fibroblasts after the indicated cycles. Cells were either mock-treated (vehicle, DMSO) or treated with different cycle lengths of 0.06 µM FTI for 1 day followed by 3 days of 1.0 µM SFN, as indicated. Single-drug treatment of 0.06 µM FTI and 1.0 µM SFN for the corresponding cycle times (4 days, 8 days, or 16 days) were carried out. Cells were fed daily. **B.** Autophagy activity of the same cells and culture conditions as in (A) was measured using monodansylcadaverine (MDC). Data are expressed as the mean ± S.D. (*p-value ≤ 0.05; *n* = 5). **C.**, **D.**, and **E.** Representative Western blots of A-type lamins in control and HGPS fibroblasts. Cells were treated in cycles of 1 day of 0.06 µM FTI treatment followed by 3 days of 1.0 µM SFN. The length of the cycles is indicated above each Western blot. In parallel, single-drug treatments of 0.06 µM FTI or 1.0 µM SFN for the corresponding cycle durations were performed. Blots were probed with prelamin A, lamin A/C, and b-actin antibodies (*n* = 4). Numbers above the prelamin A band indicate the fold-change of prelamin A relative to their mock-treated counterparts. **F.**, **G.**, and **H.** The ratios of A-type lamins were determined within each sample analyzed by Western blotting with lamin A/C antibodies (C, D, and E). Data are presented as the ratio ± S.D. (*p-value ≤ 0.05; *n* = 5). **I.** Population doublings were calculated as stated in Materials and Methods for control and HGPS fibroblasts that were either mock-treated (vehicle, DMSO) or treated with different cycle lengths of 0.06 µM FTI for 1 day followed by 2 days of 1.0 µM SFN, as indicated. Single-drug treatment of 0.06 µM FTI or 1.0 µM SFN for the corresponding cycle times (3 days, 9 days, or 18 days) was performed. Cells were fed daily. **J.** The same cells and culture conditions as in (I) were used to measure autophagy as described in the Methods. Data are expressed as the mean ± S.D. (*p-value ≤ 0.05; *n* = 5). **K.**, **L.**, and **M.** Representative Western blots of A-type lamins in control and HGPS fibroblasts. Cells were mock-treated or treated with different cycles of 1 day of 0.06 µM FTI followed by 2 days of 1.0 µM SFN, as indicated. Single-drug treatments of 0.06 µM FTI or 1.0 µM SFN for the corresponding times were performed in parallel. Blots were probed with prelamin A, lamin A/C, and b-actin antibodies (*n* = 4). The fold-change of prelamin A is indicated above each prelamin A band and is relative to the mock-treated counterparts. (N, O, and P) The ratios of A-type lamins were determined within each sample analyzed by Western blotting with lamin A/C antibodies **N.**, **O.**, and **P.** Levels are presented as the ratio ± S.D. (*p-value ≤ 0.05; *n* = 5).

To circumvent the drug toxicity of FTI and SFN when applied together, we established a new treatment regimen consisting of a treatment cycle with each drug administered separately. To develop this treatment regimen, we took advantage of the following previous findings: (1) FTI treatment is reversible and can be rapidly washed out from cells within 3 days after FTI treatment cessation [28]; and (2) A significant level of progerin clearance is observed in HGPS cells treated with 1 µM SFN for a period of 3 days [20]. Based on these observations, we chose a regimen in which cells were treated with 0.06 µM FTI alone for 1 day followed by daily treatment with 1 µM SFN alone for a period of 3 days (hereafter, 4-day regiment; Figure 3A-3H). The regimen was applied either once, or successively 2-4 times, and different functional parameters were measured. First, we determined the population doubling in cultures treated with the individual drugs or treated intermittently with FTI and SFN individually for 1, 3 and 4 cycles of 4-day regimen (Figure 3A). Mock-treated control cells grew faster than mock-treated HGPS cells (Figure 3A) as reported previously [20]. Normal cells exposed to FTI alone showed decreased growth, while HGPS cells stopped growing after 8 days of treatment (Figure 3A). In contrast, the growth rate was increased in both normal and HGPS cultures treated with SFN alone or intermittent FTI and SFN treatment (4-day regimen) (Figure 3A). The basal levels of autophagy were lower in HGPS than in control cells (Figure 3B), [20]. FTI treatment alone stimulated autophagy levels as reported previously [29, 30]. Autophagy levels were also increased in both control and HGPS cells by SFN alone, and by the 4-day regimen (Figure 3B).

Since autophagy levels were elevated by all treatment regimens, we performed Western blot analysis to quantify the ratio of A-type lamins (Figure 3C-3H). To track the efficacy of FTI treatment, we monitored the levels of prelamin A, which is known to accumulate proportionally to the levels of farnesyltransferase inhibition [31] (Figure 3C-3E). As expected, FTI treatment alone induced high levels of prelamin A accumulation in both control and HGPS cells (Figure 3C-3E). However, cells treated with SFN alone, or the 4-day regimen showed little to no signal for prelamin A (Figure 3C-3E). This indicated that during the 3 days of SFN treatment in the 4-day regimen, FTI was efficiently washed out from the cells and the prelamin A that had accumulated was rapidly processed in both control and HGPS cells.

In nearly all assays, the 4-day regimen produces results that are similar to treatment with SFN alone. SFN treatment induced a 7% reduction of progerin levels at day 4, 16% at day 8, and 20% at day 16. While the 4-day treatment regimen resulted in a similar reduction at day 4 (7%), the progerin levels were further decreased at days 8 (20%) and 16 (25%) (Figure 3C-3E). Concomitantly with the progerin reduction, the ratio of A-type lamins was ameliorated in the HGPS cells (Figure 3C-3E). However, the long-term 4-day regimen was more efficient in enhancing progerin clearance and correcting A-type lamin ratios than SFN alone. Treatment of the HGPS cells with FTI alone was less efficient in reducing progerin levels (3% at day 4, 11% at day 8, and 14% at day 16) and induced prelamin A build-up and therefore did not ameliorate the A-type lamin ratios (Figure 3C-3E). These findings indicate that the 4-day regimen improved HGPS cell proliferation, enhanced autophagy, increased progerin clearance and prevented prelamin A accumulation, which jointly ameliorated the A-type lamin status in HGPS cells.

To test the possibility that shortening the intermittent FTI/SFN regimen to 3 days (3-day regimen) could further enhance progerin clearance, we investigated the same parameters (Figure 3I-3P). One cycle of the 3-day regimen induced growth rates similar to those of SFN treatment alone in both the control and HGPS cells (Figure 3I). However, comparison of the growth rates between the 3-day and 4-day regimens demonstrated that the 4-day regimen induced 6% higher growth rates after one cycle, and the same trend was observed with increasing numbers of treatment cycles (Figure 3A, 3I). Four cycles of the 4-day regimen resulted in 14% higher proliferation rates than 6 cycles of the 3-day regimen.

Next, we compared autophagy activation between the 3- and 4-day regimens (Figure 3B and 3J). Autophagy levels were 13% higher following 1 cycle of 4-day regimen compared with 1 cycle of 3-day regimen. Similarly, 2 cycles of 4-day regimen induced 20% higher autophagy levels than 3 cycles of 3-day regimen (Figure 3B and 3J). Likewise, 4 cycles of 4-day regimen induced a 13% autophagy increase compared with 6 cycles of 3-day regimen. Western blot analysis also revealed that the 3-day regimen was less efficient in clearing progerin compared with the 4-day regimen (Figure 3C-3H and 3K-3P). Additionally, long-term treatment with the 3-day regimen was less efficient at preventing prelamin A accumulation (Figure 3K, 3L and 3M). These findings indicate that the 4-day regimen was most efficiently ameliorated proliferation and autophagy, reduced progerin levels and prevented prelamin A accumulation in HGPS cells. “

### Treatment cycles with FTI/SFN improve the ROS and ATP status in HGPS cells

**Figure 4:**
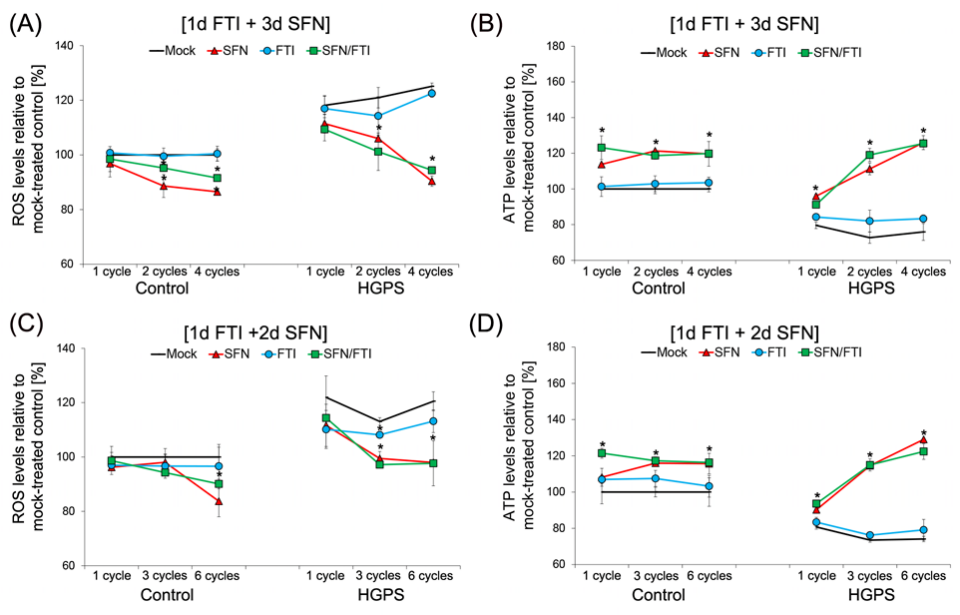
Intermittent treatment cycles with 1 day of FTI followed by 3 days of SFN improve ROS and ATP levels in HGPS cells **A.** Intracellular ROS levels were determined by measuring oxidized dichlorofluorescein (DCF) levels as described in the Methods. Control and HGPS cells were mock-treated or treated with different number of cycles of 1d of 0.06 µM FTI followed by 3d of 1.0 µM SFN as indicated. Single-drug treatments of 0.06 µM FTI or 1.0 µM SFN for the corresponding cycle times were performed. Data represents the mean percentage ± S.D. (*p-value ≤ 0.05; *n* = 5) relative to mock-treated controls. **B.** Cellular ATP levels of mock-treated or treated control and HGPS cells, as described in (A), were measured using a CellTiter-Glo assay as described in the Methods. Data are expressed as the mean percentage ± S.D. (*p-value ≤ 0.05; *n* = 5) relative to mock-treated control cells. **C.** Control and HGPS cells were either mock-treated or treated with different cycles of 1d of 0.06 µM FTI followed by 2d of 1.0 µM SFN to determine intracellular ROS levels, as described in the Methods. Single-drug treatment of 0.06 µM FTI or 1.0 µM SFN was performed in parallel for the corresponding cycle times. Data are expressed as the mean percentage ± S.D. (*p-value ≤ 0.05; *n* = 5) relative to mock-treated controls. **D.** The same cells as in (C) were used to measure cellular ATP levels, as described in the Methods. Data are presented as the mean percentage ± S.D. (*p-value ≤ 0.05; *n* = 5) relative to mock-treated control cells.

Previous studies have shown that HGPS cells exhibit decreased basal levels of ATP and elevated levels of reactive oxygen species (ROS) [32]. We previously reported that treatment with SFN alone was efficient in ameliorating ROS and ATP levels in HGPS cells [20]. We therefore investigated whether treatment cycles with FTI/SFN in HGPS cells might further improve these two parameters (Figure 4).

We further confirmed that ROS basal levels were higher in HGPS cells compared to normal cells (Figure 4A). FTI treatment alone did not reduce ROS levels in HGPS cells compared to mock-treated HGPS cells during the different durations of treatment (Figure 4A). Treatment with SFN alone or with cycles of the 4-day regimen induced similar reductions in ROS levels in HGPS cells, which were further reduced with increased treatment time (Figure 4A).

As reported previously, HGPS cells exhibit decreased basal levels of cellular ATP levels compared to normal cells (Figure 4B). Investigating the effect of single drugs and intermittent treatment with FTI/SFN on ATP levels provided further evidence that SFN treatment induces an increase in cellular ATP that was further improved with increased treatment duration (Figure 4B). FTI treatment alone had no significant effect on ATP levels in both cells types (Figure 4B). The 4-day regimen induced an increase in ATP levels that was similar to treatment with SFN alone (Figure 4B). After 2 cycles of 4-day regimen or SFN treatment, ATP levels in the HGPS cells were much higher than the basal ATP levels of mock-treated normal cells. After 4 cycles of 4-day regimen or SFN treatment, the levels of ATP in HGPS cells remained high and at comparable levels to that observed in similarly treated normal cells (Figure 4B). Collectively, the findings indicate that 4-day regimen cycles induced a similar amelioration in ROS and ATP levels in HGPS cells as observed with SFN treatment alone.

To further assess the impact of these treatment cycle regimens, we compared the effect of the 4-day regimen (Figure 4A, 4B) with the 3-day regimen (Figure 4C, 4D) on ROS and ATP status. Both, regimens induced a decrease in ROS levels. However, 4 cycles of 4-day regimen induced 4 % lower ROS levels compared to 6 cycles of 3-day regimen in HGPS cells (Figure 4A, 4C). This indicates that the 4-day regimen was more effective in reducing ROS levels in HGPS cells. Comparing the effects of both treatment cycles on cellular ATP levels, we found that both FTI/SFN regimens induced similar increases in ATP levels in both normal and HGPS cells (Figure 4B, 4D). Together, these findings demonstrate FTI treatment alone had no effect on ROS and ATP levels, while FTI/SFN cycle treatments induced similar positive impacts on both parameters in HGPS cells as observed with SFN treatment alone. Thus, the amelioration of these two components is the result of SFN cellular action.

### Intermittent FTI/SFN treatment normalizes nuclear shape in HGPS cells and prevents prelamin A accumulation and donut-shaped nuclei formation

**Figure 5:**
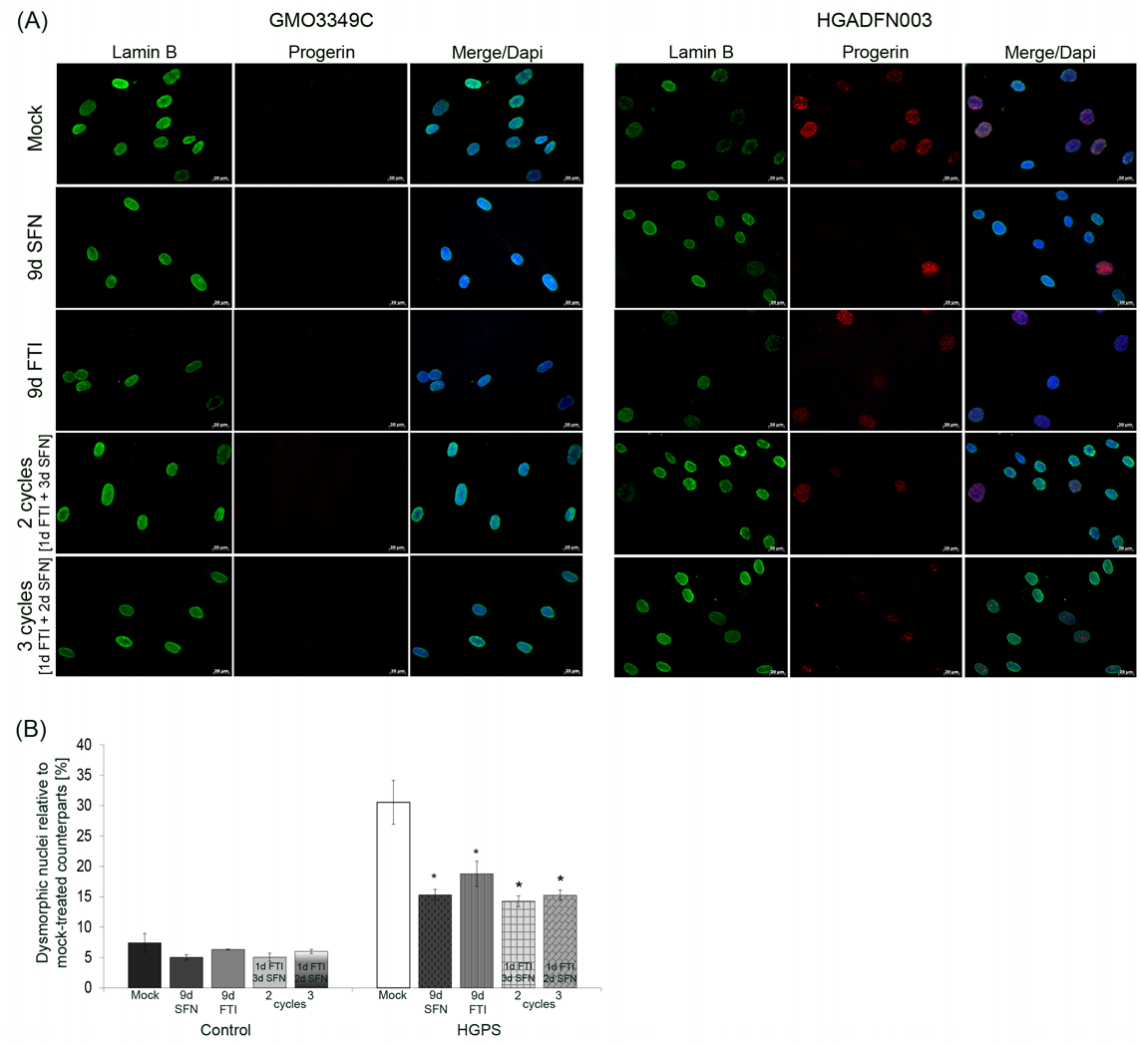
Intermittent treatment cycles with 1 day of FTI followed by 3 days of SFN reverse nuclear shape alterations in HGPS cells **A.** Immunochemistry was performed on control (GMO3349C) and HGPS (HGADFN127) fibroblasts using antibodies directed against the indicated proteins (lamin B1 and progerin) after 2 cycles of 1 day of 0.06 µM FTI and 3 days of 1.0 µM SFN treatment or 3 cycles of 1 day of 0.06 µM FTI and 2 days of 1.0 µM SFN. Single-drug treatment of 0.06 µM FTI or 1.0 µM SFN for 9 days was performed in parallel. Cells were treated daily with fresh medium. Representative images are shown (*n* = 4). Scale-bar: 20 µm. **B.** The same cells as in (A) were used to determine the frequency of misshapen nuclei (dysmorphic) by direct counts of an average of 900 nuclei (*n* = 4).

**Figure 6:**
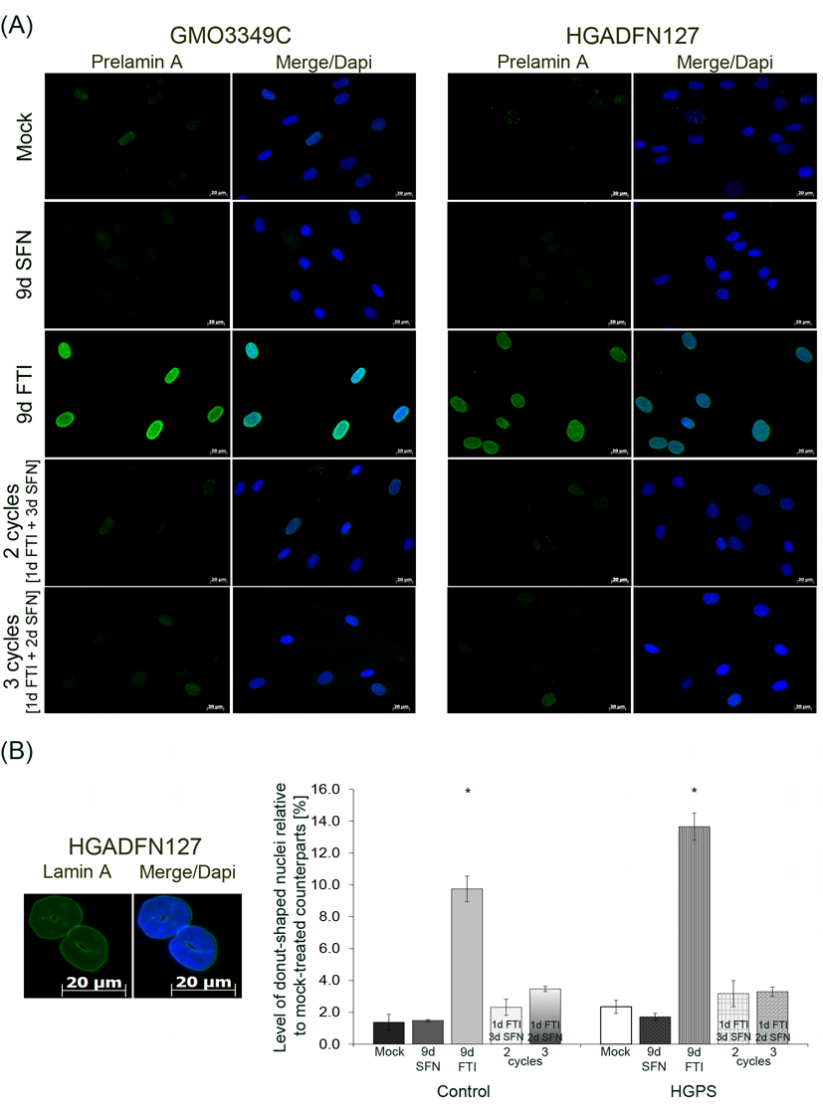
Intermittent treatment cycles with 1 day of FTI followed by 3 days of SFN prevent prelamin A accumulation and donut-shaped nuclei formation **A.** Immunochemistry was performed on control (GMO3349C) and HGPS (HGADFN127) fibroblasts using an anti-prelamin A antibody. Cells were treated for 2 cycles of 1 day 0.06 µM FTI and 3 days 1.0 µM SFN treatment or 3 cycles of 1 day 0.06 µM FTI and 2 days 1.0 µM SFN treatment. Single-drug treatment of 0.06 µM FTI or 1.0 µM SFN for 9 days was performed in parallel. Representative images are shown (*n* = 4). Scale bar: 20 µm. **B.** The same cells and conditions as in (A) were used to analyze donut-shaped nuclei by direct counts of lamin A-stained nuclei. An average of 1,000 nuclei were counted. Left panel shows representative image of nuclei that were considered donut-shaped nuclei. Right panel shows the quantification of donut-shaped nuclei relative to mock-treated control. Data are presented as the percentage ± S.D. (*p-value ≤ 0.05; *n* = 3).

HGPS cells exhibit dysmorphic nuclei and reduced levels of nuclear components such as lamin B1 [7, 33]. FTI treatment has been shown to normalize nuclear envelope abnormalities [34] but also disrupt the lamin B meshwork, induce prelamin A accumulation and the formation of donut-shaped nuclei [5, 16, 20]. Therefore, we investigated the impact of the intermittent FTI/SFN treatment regimens on progerin, lamin B1 distribution as well as nuclear envelope morphology (Figure 5 and Figure 6). Single-drug treatment with FTI, SFN or vehicle was performed in parallel for comparison.

In mock-treated HGPS cells, weak lamin B1 staining was observed in brightly progerin-positive nuclei (Figure 5A). In SFN-treated and 4-day regimen-treated HGPS cells, the progerin signal was overall reduced, and the presence of dim lamin B1-stained nuclei was also decreased as reported previously [20]. In FTI-treated cells, lamin B1 and progerin signals were similar to mock-treated cells (Figure 5A). Lamin B1 signal was similar in cells treated with SFN alone or 4-day regimen cycles (Figure 5A). Progerin signal was reduced in cells treated with 4-day regimen, and the number of brightly progerin-positive nuclei was decreased (Figure 5A). Collectively, the progerin signal was reduced in cells treated with SFN alone or the 4-day regimen.

Scoring the number of dysmorphic nuclei in HGPS cells treated with FTI or SFN for nine days or with the 4-day regimen revealed that their numbers were significantly reduced (Figure 5B).

Next, we analyzed the distribution and presence of prelamin A after these different drug treatments (Figure 6A). In accordance with previous studies, prelamin A was detected in both cell types treated with FTI alone (Figure 6A). The prelamin A signal was barely detected in cells treated with the 4-day regimen or with SFN alone (Figure 6A). This indicated that effective prelamin A maturation occurred during the period of SFN treatment in the 4-day regimen. Previous studies have shown that FTI treatment induced an increase in the formation of donut-shaped nuclei [20, 35]. We quantified the frequency of donut-shaped nuclei after FTI treatment, SFN and intermittent FTI/SFN regimens in both control and HGPS cells (Figure 6B). Indeed, high numbers of donut-shaped nuclei were observed in both normal (9 %) and HGPS (12 %) cells treated with FTI for 9 days. In contrast, the number of donut-shaped nuclei remained low in cells treated with intermittent FTI/SFN cycles (Figure 6B). Thus, intermittent treatment with FTI/SFN prevented the accumulation of these abnormal nuclei in both normal and HGPS cells.

### Intermittent FTI/SFN treatment cycles reduce DNA damage levels in HGPS cells

**Figure 7:**
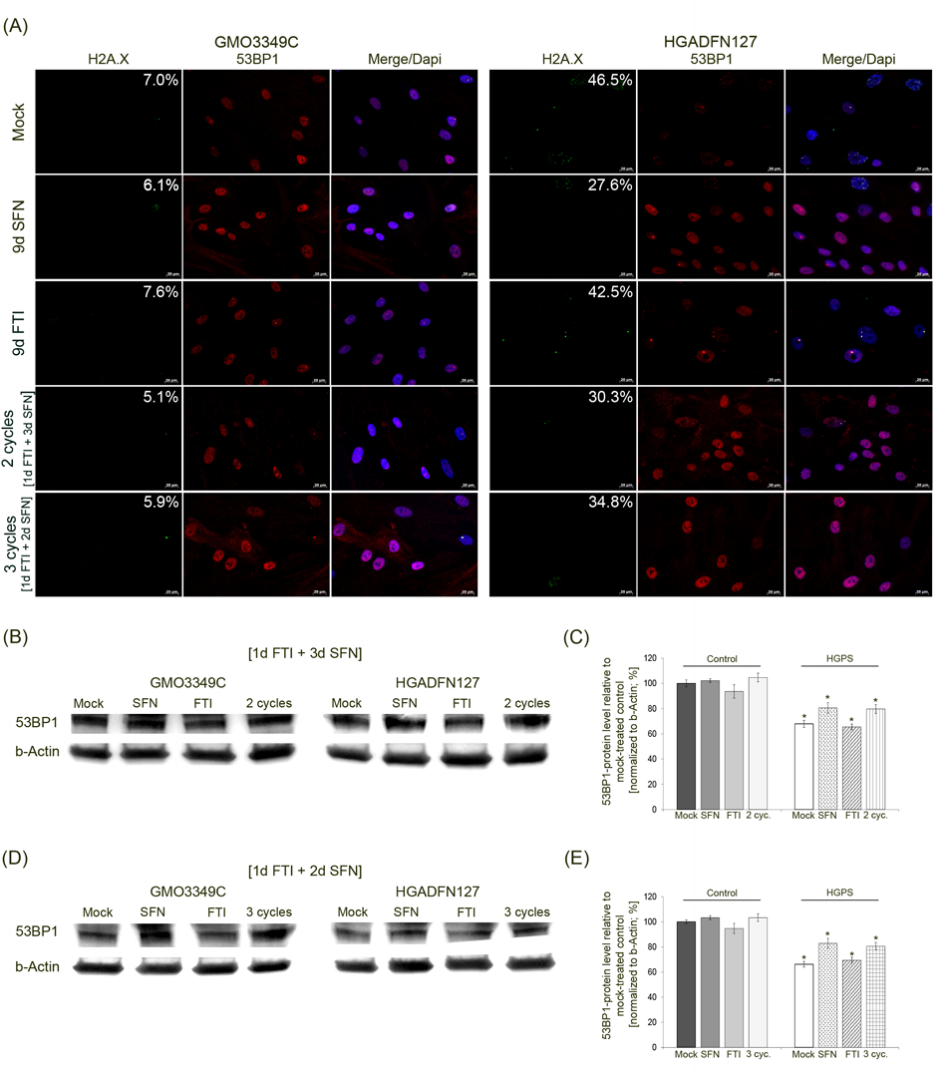
Intermittent treatment cycles with 1 day of FTI followed by 3 days of SFN improve 53BP1 levels in HGPS cells **A.** Immunochemistry was performed on control (GMO3349C) and HGPS (HGADFN127) fibroblasts either mock-treated or treated with the 1d 0.06 µM FTI/3d 1.0 µM SFN regimen for 2 cycles or the 1d 0.06 µM FTI/2d 1.0 µM SFN regimen for 3 cycles. In parallel, cells were treated with single-drug treatments of 0.06 µM FTI or 1.0 µM SFN for 9 days. Antibodies against γH2A.X and 53BP1 were used. Representative images are shown (*n* = 4). Numbers indicate the percentage of cells with DNA damage. Scale bar: 20 µm. **B.** Western blot analysis of control (GMO3349) and HGPS cells (HGADFN127) treated for 2 cycles of the 1d 0.06 µM FTI/3d 1.0 µM SFN regimen. Single-drug treatment of 0.06 µM FTI or 1.0 µM SFN for 8 days in control and HGPS cells was performed in parallel. Antibodies against 53BP1 and b-Actin were used. Representative images are shown (*n* = 3). **C.** Quantification of 53BP1 levels from panel (B). Levels were normalized to b-actin and presented as the percentage ± S.D. relative to mock-treated control cells (*p-value ≤ 0.05; *n* = 3). **D.** Western blot analysis of control (GMO3349) and HGPS cells (HGADFN127) that were treated as described in (A) for 3 cycles of the 1d 0.06 µM FTI/2d 1.0 µM SFN regimen. Single-drug treatments of 0.06 µM FTI or 1.0 µM SFN was performed in parallel for 9 days. Antibodies against 53BP1 and b-Actin were used. Representative images are shown (*n* = 3). **E.** Quantification of 53BP1 levels from panel (D). Levels were normalized to b-actin and presented as the percentage ± S.D. relative to mock-treated control cells (*p-value ≤ 0.05; *n* = 3).

HGPS fibroblasts are known to accumulate DNA damage due to a defective DNA damage repair response [36]. To investigate whether the intermittent FTI/SFN treatment could ameliorate this defect in HGPS cells, we performed a series of immunofluorescence detections on control and HGPS cells treated with SFN, FTI, or intermittent FTI/SFN treatment cycles (Figure 7). Scoring the frequency of nuclei harboring the DNA damage marker γH2A.X indicated that the average number of nuclei harboring γH2A.X foci was 47 % in mock-treated HGPS cells compared to 7 % in mock-treated control cells (Figure 7A). Treatment with SFN alone led to a significant reduction of DNA damage levels in HGPS cells (27 %) after 9 days. FTI treatment alone showed no significant changes in DNA damage levels in control or HGPS cells (Figure 7A). After 2 cycles of the 4-day regimen (treatment period of 8 days), the level of DNA damage was reduced to an average of 30 % in HGPS cells. After 3 cycles of the 3-day regimen (treatment period of 9 days), the number of HGPS nuclei harboring DNA damaged was reduced to 34.8 %. This indicated that the 4-day regimen was more efficient in reducing DNA damage levels in HGPS cells.

In accordance with previous studies, localization of the DNA damage repair factor 53BP1 revealed that its expression in mock-treated HGPS cells is lower compared to normal cells (Figure 7B) [20]. Cells treated with SFN alone or the 4-day regimen showed increased signals for 53BP1 in both control and HGPS cells (Figure 7A). The distribution of 53BP1 in those cells was more homogeneous throughout the nucleoplasm. In contrast, mock-treated HGPS cells exhibited 53BP1 aggregates in some nuclear areas, and the 53BP1 signal was overall weaker. After FTI treatment alone, the 53BP1 signal was weakened in both control and HGPS cells (Figure 7A).

Western blot analysis of 53BP1 in cells treated with the different regimens indicated that, its levels were indeed reduced in HGPS cells relative to normal cells (Figure 7B, 7C). 53BP1 protein levels were increased in HGPS cells treated with SFN alone or with cycles of 4-day treatment (Figure 7B-7E).

**Figure 8:**
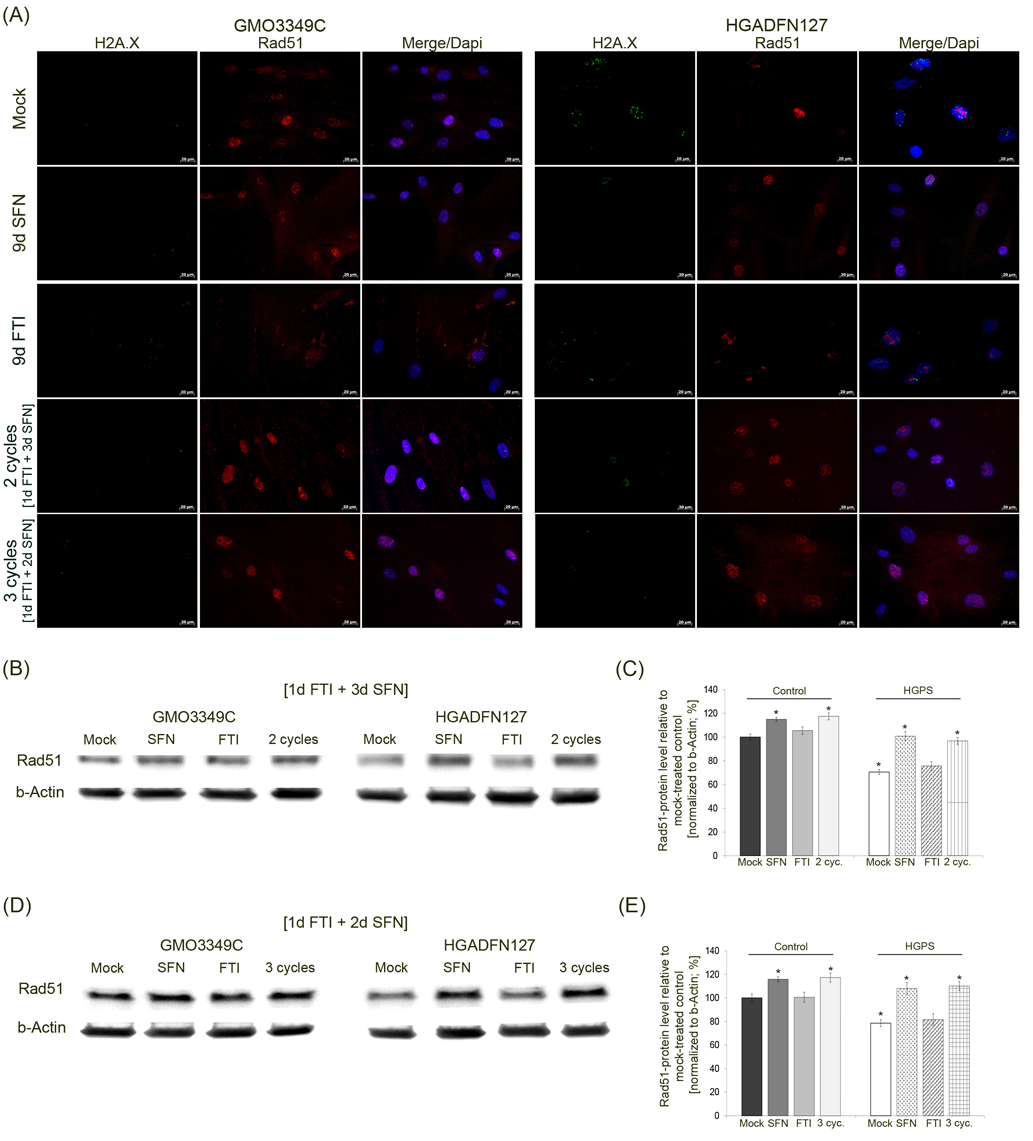
Intermittent treatment cycles with 1 day of FTI followed by 3 days of SFN ameliorate Rad51 levels in HGPS cells **A.** Immunochemistry was performed on control (GMO3349C) and HGPS (HGADFN127) fibroblasts either mock-treated or treated with 1d 0.06 µM FTI/3d 1.0 µM SFN regimen for 2 cycles or 1d 0.06 µM FTI/2d 1.0 µM SFN regimen for 3 cycles. Single-drug treatments of 0.06 µM FTI or 1.0 µM SFN for 9 days were performed in parallel. Cells were immunolabeled with antibodies against γH2A.X and Rad51. Representative images are shown (*n* = 4. Scale bar: 20 µm. **B.** Western blot analysis of control (GMO3349) and HGPS cells (HGADFN127) treated for 2 cycles of 1d 0.06 µM FTI/3d 1.0 µM SFN regimen. Single-drug treatments of 0.06 µM FTI or 1.0 µM SFN for 8 days were performed in parallel. Blots were probed with antibodies against Rad51 and b-Actin. Representative image is shown (*n* = 3). **C.** Quantification of Rad51 levels from panel (B). Levels were normalized to b-actin and presented as the percentage ± S.D. relative to mock-treated control cells (*p-value ≤ 0.05; *n* = 3). **D.** Western blot analysis of control (GMO3349) and HGPS cells (HGADFN127) were treated as described in (A) for 3 cycles of 1d 0.06 µM FTI/2d 1.0 µM SFN regimen. Single-drug treatments of .06 µM FTI or 1.0 µM SFN were carried along for 9 days. Blots were probed with antibodies against Rad51 and b-Actin. Representative image is shown (*n* = 3). **E.** Quantification of Rad51 levels from panel (D). Levels were normalized to b-actin and presented as the percentage ± S.D. relative to mock-treated control cells (*p-value ≤ 0.05; *n*= 3).

In addition to the status of 53BP1, we investigated Rad51, another DNA damage repair factor that activates homologous recombination (Figure 8A). The levels of Rad51 were decreased in mock-treated HGPS cells compared to their control counterparts (Figure 8B). Immunofluorescence staining of Rad51 revealed some bright foci in HGPS cells that were not co-localized with γH2A.X foci (Figure 8A). After SFN and FTI/SFN intermittent treatment cycles, HGPS cells showed an increase in Rad51 signal throughout the nucleus and at sites of γH2A.X foci as observed in mock-treated normal cells. After FTI treatment, Rad51-stained foci were not co-localized with γH2A.X foci in HGPS cells (Figure 8A). Rad51 protein levels were increased in HGPS cells treated with SFN alone or with cycles of FTI/SFN regimens (Figure 8B-8E). Collectively, our findings indicate that treatment with SFN alone or with FTI/SFN intermittent treatment cycles efficiently raised the levels of both 53BP1 and Rad51, thus improving DNA damage repair processes in HGPS cells.

## DISCUSSION

Our results support the hypothesis that exploiting the pharmacological properties of the farnesyltransferase inhibitor, lonafarnib and sulforaphane are promising for HGPS management due to their abilities to improve the HGPS cellular phenotype [20, 37]. Our findings also demonstrate that intermittent treatment with FTI and SFN when administered separately and in cycles improve HGPS cellular homeostasis, while combined treatment with both drugs induces cytotoxicity.

To date FTI (Ionafarnib) has been used in clinical trials for children with HGPS [38]. SFN preparations are currently used in several clinical trials, including a phase II trial for children with autism (clinicaltrials.gov). FTI and SFN have not yet been administrated together in animal models or in human. Therefore, using HGPS cell based model, we tested the effects of FTI and SFN treatment on HGPS cellular phenotype in hopes to identifying a synergistic positive effect and a restoration of cellular function. We postulated that FTI would delocalize progerin from the nuclear envelope by blocking progerin farnesylation and thereby increase its turnover rate [19]. This should aid in the clearance of progerin by SFN-stimulated autophagy, and through the antioxidant activities of SFN, mitochondria dysfunction should be reversed and DNA damage should be reduced in HGPS cells [20].

In accordance with our previous study, SFN administration alone induced similar beneficial effects on HGPS cell phenotype [20]. FTI administration alone induced a partial inhibition of farnesylation as indicated by the accumulation of prelamin A and improved nuclear morphology. However, the DNA damage remained and increased numbers of donut-shaped nuclei were observed [18, 20, 35, 39].

In this current study, we showed for the first time that FTI administration activated autophagy in HGPS cells. Autophagy induction by FTI was previously shown in cancer cells and its activation increased in an FTI-dose-dependent manner as shown in this study and previous studies [40, 41]. The molecular mechanism underlying FTI-autophagy induction was suggested to be the consequences of inactivating the Rheb-mTOR signaling pathway, which is a master regulator of autophagy [41]. Rheb is a farnesyltransferase substrate and an activator of mTOR. FTI, by blocking Rheb farnesylation, would prevent its intracellular localization and therefore its interaction with mTOR, which consequently would lead to the activation of autophagy. We report that HGPS and normal cells treated with FTI and SFN together and even at very low concentrations exhibit decreased growth rate and increased cell death signifying that this regimen was cytotoxic. Moreover, treatment with both drugs induced much higher levels of autophagy than treatment with each drug separately. This finding indicates that FTI and SFN exerted a synergistic and additive positive effect on autophagy activity when administered together. We therefore suggest that combined drugs over-activate autophagy, presumably reaching levels that become lethal to the cells [42, 43], but this remains to be further investigated.

To circumvent this problem, we took advantage of the reversibility of FTI, which can be cleared from cells within 2 to 3 days after its withdrawal from the culture media [28]. Based on this, we established a novel intermittent treatment regimen consisting of repeated cycles of 1-day treatment with FTI alone followed by 3 day of treatment with SFN alone. This 4-day regimen induced similar positive effects in HGPS cells as that observed with SFN treatment alone [20]. The advantage of the 4-day regimen over SFN treatment alone is its higher efficacy (5 %) in enhancing progerin clearance via autophagy. Since the build-up of progerin in the nuclear compartment is responsible for the setting of the numerous functional defects that characterize HGPS cells, the extent to which progerin levels can be reduced will have a direct impact on the degree of HGPS cells recovery.

In conclusion, this novel intermittent treatment cycle with FTI and SFN ameliorated the HGPS cellular phenotype *in vitro*, as evidenced by the numerous functional parameters investigated in this study and the enhanced progerin clearance. Our findings, however, suggests a risk of combining FTI with drugs that activate autophagy, as they can have a synergistic and additive effect on autophagy levels, which could ultimately cause cytotoxicity. Herein, we show that FTI can be administered with other drugs activating autophagy, but it requires an intermittent treatment regimen.

Ionafarnib is thus far the only treatment that has demonstrated some clinical ameliorations in children with HGPS and an increased survival of ∼1.6 years [44]. Although this improvement remains minimal, the question of whether future therapies have to be combined with FTI administration needs to be addressed, and the decision-making might not be easy for the families. Therefore, imminent clinical trials testing novel drugs and combinations of drugs should include measurements of autophagy and autophagy flux to test for unforeseen side effects of combined drug treatments. We propose that intermittent treatment cycles with FTI followed by SFN administration might be a novel therapeutic strategy for children with HGPS.

## MATERIALS AND METHODS

### Cell culture and drug treatments

Fibroblast lines (HGADFN003, HGADFN127, HGADFN155, and HGADFN164) derived from patients with HGPS were obtained from the Progeria Research Foundation Cell and Tissue Bank (www.progeriaresearch.org). Control fibroblasts (GM01651C, GM0323B, GM03349C, GM03348E, and GM08398A) were obtained from the Coriell Institute for Medical Research (Camden, NJ, USA). Cells were cultured as described previously [45]. Briefly, cells were cultured at 37 °C and 5 % CO_2_ in DMEM medium (supplemented with 15 % FBS, 1 % glutamine, 1 % PenStrep. All experiments were performed on at least 3 control and 3 HGPS primary fibroblast lines at passages 10 to 16 that have been cultured and treated in parallel to permit comparisons between control and HGPS experimental investigations.

For drug treatment experiments, sulforaphane (SFN, Sigma-Aldrich) was added to the medium at a concentration of 0.25 µM and 1.0 µM. FTI Ionafarnib (Merck, USA) was used at the following concentrations: 0.06 µM, 0.25 µM, 0.5 µM, 1.0 µM, and 1.5 µM. For combined drug treatments, SFN and FTI were added together to the medium at indicated concentrations. For intermittent drug treatments, drugs were added to the medium separately, and the culture medium was changed daily with the indicated treatment. Mock-treated fibroblasts were cultured in parallel with media containing the vehicle (DMSO).

### Population doubling determination

Control and HGPS cells were seeded in triplicate at a known density per 10 cm dish and cultured in DMEM high glucose medium for the indicated periods. Cells were harvested, and the number of cells was measured with a CASY Cell Counter (Roche, Penzberg, Germany). The numbers of cumulative population doublings (CPDs) were determined using the following formula: *n* = 3.32 (log Xh - log Xb) + S, where n is the total number of CPDs, Xh is the number of cells seeded, Xb is the number of cells harvested and S is the starting CPD as described [46].

### Western blot analysis

Cell pellets were resuspended in Laemmli sample buffer (BioRad), and Western blots were performed as described previously [45]. The membranes were incubated with the following primary antibodies: anti-lamin A/C (Thermo Fisher, 1/5000), anti-prelamin A (Santa Cruz, 1/3000), anti-53BP1 (Bethyl, 1/3000), anti-Rad51 (NBP2-32622, Novus Biological, 1/1000), anti-Lamp2 (Santa Cruz, 1/1000), anti-p62 (NovusBio, 1/3000), anti-LC3B (Sigma-Aldrich, 1/10000), and anti-β-actin (Sigma-Aldrich, 1/10000). Afterwards, the membranes were incubated with a corresponding secondary antibody coupled to horseradish peroxidase (Jackson ImmunoResearch Laboratories). Proteins were visualized with a chemiluminescence detection system (ECL substrate; BioRad) and signals were analyzed using the IMAGE LAB software (BioRad). Protein signals were quantified by normalizing to β-actin, as indicated.

### Immunocytochemistry

For immunocytochemistry, fibroblasts were grown directly on coverslips. Cells were fixed in 100 % methanol at -20 °C for 10 min. Subsequently, cells were further processed for immunohistochemistry as previously described [45]. The following primary antibodies were used: anti-progerin S9 (1 µg mL^-1^) [47], anti-lamin A (Abcam, 1/500), anti-lamin B1 (Santa Cruz Biotechnology, 1/75), anti-γH2AX (Millipore, 1/200), anti-53BP1 (A300-272A, Bethyl, 1/1000), and anti-Rad51 (Novusbio, 1/300). The secondary antibodies were affinity-purified Alexa Fluor 488 goat or donkey IgG antibodies (Molecular Probes) and Cy3-conjugated IgG antibodies (Jackson ImmunoResearch). DAPI in Vectashield mounting medium (Vector Inc.) was used to counterstain the samples. Images were acquired by using an Axioplan fluorescence microscope (Carl Zeiss).

### Autophagy measurements in fibroblasts

Autophagic vacuoles were determined in control and HGPS cells using an Autophagy/Cytotoxicity Dual Staining Kit (Cayman Chemical Company). Mock-treated and drug-treated cells were harvested and seeded in triplicate at the same cell density on a 96-well plate. Cells were allowed to attach overnight at culture conditions. Afterwards, the plate was centrifuged and 100 µL of monodansylcadaverine (MDC) was added to the wells at a 1:1000 ratio. After incubation at 37 °C for 10 min, the plate was centrifuged and washed with assay buffer. An excitation wavelength of 335 nm and an emission wavelength of 512 nm were used to measure the autophagy levels with POLARstar OMEGA (BMG Labtech). For live-cell immunochemical staining, cells were grown directly on coverslips and allowed to attach overnight. Then, propidium iodide (1/1000) was added for 2 min at room temperature. Coverslips were washed twice with Assay buffer before monodansylcadaverine was added at a ratio of 1/1000 followed by an incubation step of 10 min at 37 °C. Finally, coverslips were washed with assay buffer and then analyzed using an Axioplan fluorescence microscope (Carl Zeiss).

### Measurement of ROS in fibroblasts

The quantity of reactive oxygen species was measured using a Cellular ROS Detection Assay Kit from Abcam. Mock-treated and drug-treated cells were seeded in triplicate at the same density on a 96-well plate and were allowed to attach overnight. Adherent cells were incubated with 25 µL of DCFDA for 45 min at 37 °C, washed and measured. Fluorescence was measured with POLARstar OMEGA (BMG Labtech) at an excitation wavelength of 355 nm and an emission wavelength of 512 nm.

### Measurements of ATP in fibroblasts

The intracellular ATP content of treated and mock-treated fibroblasts was measured using a CellTiter-Glo Luminescent Cell Viability Assay (Promega). Cells were harvested and seeded at equal densities in triplicate in 96-well plates. These cells were allowed to attach at 37 °C for 12 hours and were counted before conducting the assays. Cells were then incubated with 100 µL of CellTiter-Glo reagent (CellTiter-Glo buffer plus CellTiter- Glo substrate) for 10 min, and the luminescence intensity was measured. An ATP standard was assessed in parallel.

### Statistical analyses

For all the experiments, the results are presented as the mean ± SD. Comparisons were performed using Student’s t-test. A p-value of p ≤ 0.05 was considered statistically significant. Sample sizes are indicated in the figure legends.

## SUPPLEMENTARY MATERIALS FIGURES


