# Supplementary material for: Intermittent treatment with farnesyltransferase inhibitor and sulforaphane improves cellular homeostasis in Hutchinson-Gilford progeria fibroblasts
Source: Oncotarget. 2017 Jul 18;8(39):64809–26. doi: 10.18632/oncotarget.19363 (PMC5630293; doi:10.18632/oncotarget.19363)
Supplement: Supplementary file 1 [file oncotarget-08-64809-s001.pdf]

Intermittent treatment with farnesyltransferase inhibitor and sulforaphane improves cellular homeostasis in Hutchinson-Gilford progeria fibroblasts

SUPPLEMENTARY MATERIALS

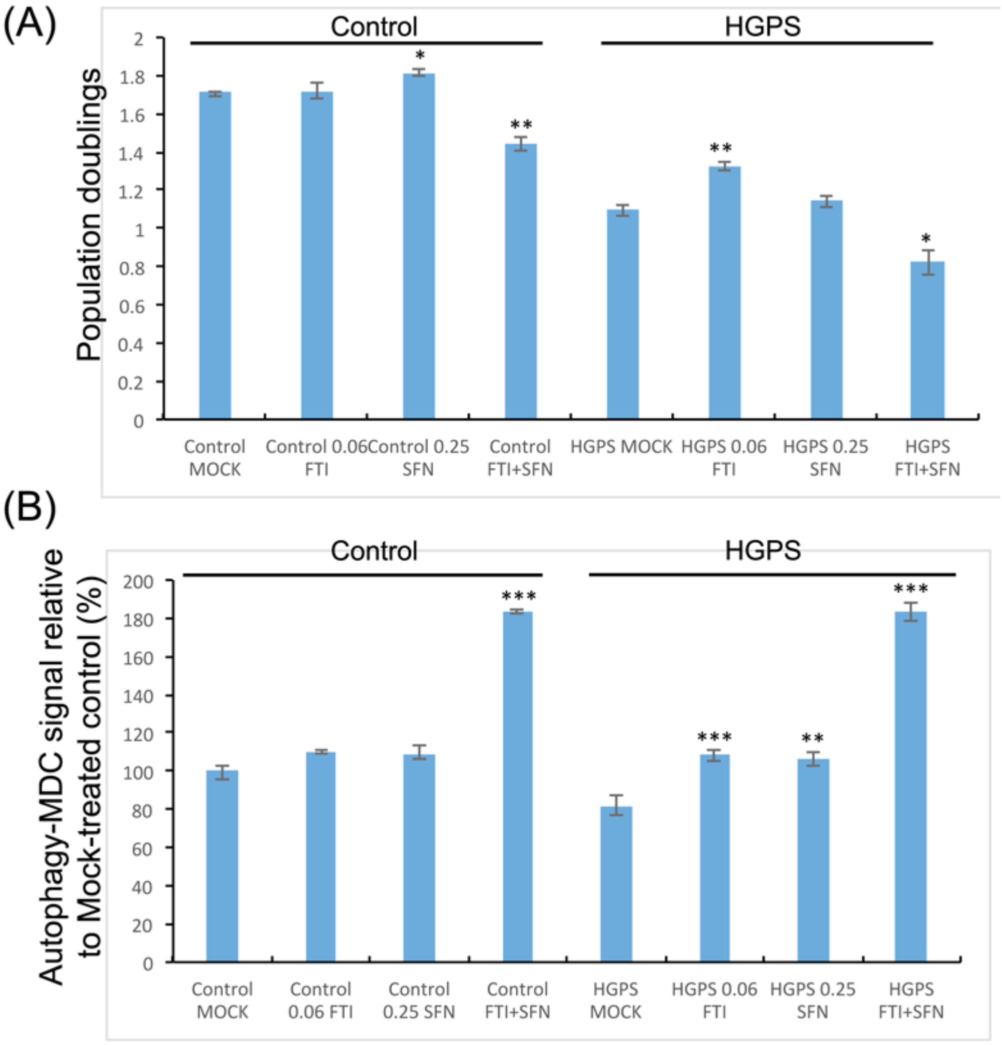

Supplementray Figure 1: Effect of combining 0.06 µM of FTI with 0.25 µM of SFN on HGPS cells. (**A**) Population doublings were calculated as stated in Materials and Methods for control and HGPS fibroblasts. Cells were either treated with vehicle (DMSO), SFN (0.25 µM), FTI (0.06 µM), or combined drugs at the following concentrations: 0.06 µM FTI plus 0.25 µM SFN for a period of 3 days. (**B**) The same cells as in (A) were used to measure the levels of autophagy vacuoles by monodansylcadaverine (MDC) as described in Materials and Methods. Data are expressed as the mean ± S.D. (*p-value ≤ 0.05; n=3).

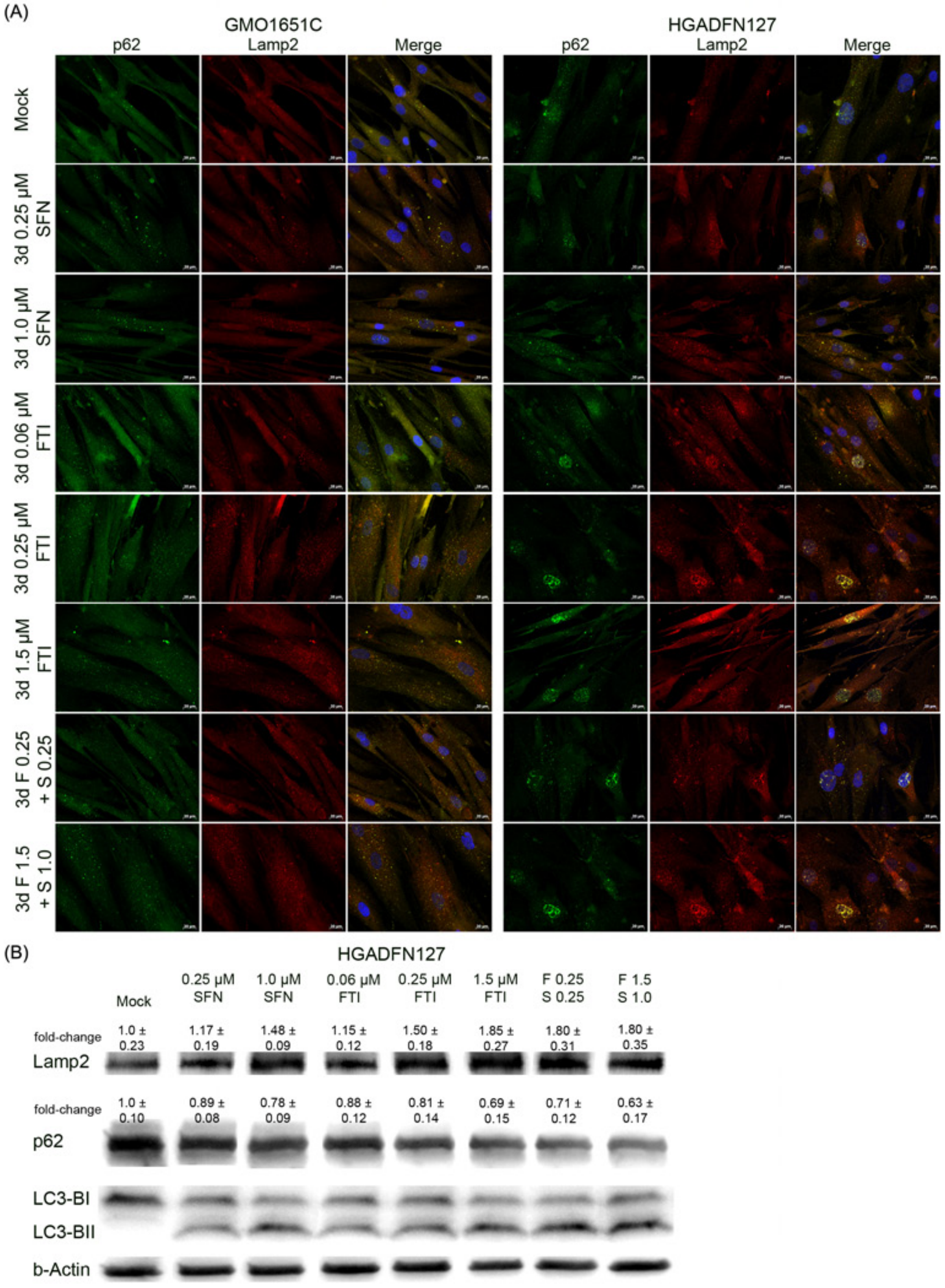

Supplementary Figure 2: Analysis of autophagic flux in HGPS cells treated with FTI and SFN in combination. (**A**) Immunochemistry was performed of control (GMO1651C) and HGPS (HGADFN127) cells using antibodies against p62 and LAMP2. Cells were either mock-treated or treated with 0.25 μM SFN, 1.0 μM SFN, 0.06 μM FTI, 0.25 μM FTI, 1.5 μM FTI, or the combination of both drugs at 0.25 μM SFN/0.25 μM FTI, or 1.0 μM SFN/1.5 μM FTI for a period of 3 days, as indicated. Representative images are shown (n=3). Scale bar: 20 μM. (**B**) Western blot analysis of the same samples as in (A) was performed using antibodies against Lamp2, p62, LC3B, or b-Actin. Numbers above each band indicate the fold-change ± S.D. of protein expression relative to their mock-treated counterparts. Representative image are shown (n=3).
